# Creation and Validation of a Temperature-Based Phenology Model for *Meloidogyne incognita* on Common Bean

**DOI:** 10.3390/plants10020240

**Published:** 2021-01-26

**Authors:** Ariadna Giné, Patricia Monfort, Francisco Javier Sorribas

**Affiliations:** Agri-Food Engineering and Biotechnology Department, Universitat Politècnica de Catalunya, Esteve Terradas 8, 08860 Castelldefels, Barcelona, Spain; patricia.monfort.1@gmail.com

**Keywords:** base temperature, degree days, *Phaseolus vulgaris*, root-knot nematodes, soil temperature, thermal requirements

## Abstract

The thermal requirements of *Meloidogyne incognita* on *Phaseolus vulgaris* in a set of constant soil temperatures were determined and the phenology model was validated at fluctuating soil temperatures. The base temperature (*Tb*) and the thermal constant (*S*) from nematode inoculation to females starting to lay eggs were 11.3 °C and 323 accumulated degree days (DD), respectively; *Tb* = 10.5 °C and *S* = 147 DD from egg production to emergence of juveniles; and *Tb* = 11.1 °C and *S* = 476 DD for life cycle completion. At fluctuating soil temperatures in pots with the minimum lower than *Tb* and the maximum higher than *To* (optimal temperature), the DD calculation was carried out by the average daily temperature–*Tb* (ADTb) and the single sine method over *Tb* (SSTb) with horizontal, intermediate, and vertical cutoffs. The most accurate were the ADTb and the SSTb with horizontal and intermediate cutoffs (93–106% of the predicted value) but the vertical underestimated the accumulated DD (75–82% of the predicted value). When fluctuating soil temperatures were between *Tb* and *To* in a plastic greenhouse, only the ADTb method was used. Life cycle completion was observed around 465 DD (accuracy between 0.95 and 0.99) at four different transplanting dates.

## 1. Introduction

The Mediterranean basin represents the highest concentration of horticultural production in Europe and is characterized by cropping few highly valuable crops in the majority of cases. The most frequent crops belong to the Solanaceae (mainly tomato, pepper, and aubergine) and the Cucurbitaceae (mainly cucumber, melon, zucchini squash, and watermelon) families [1]. However, in some growing areas, the rotation sequence includes members of the *Fabaceae* families, such as the common bean, *Phaseolus vulgaris* L., which is among the most cultivated legumes worldwide. In 2018, Spain cultivated ca. 7885 ha of green bean with a production of 138,925 tonnes, being the second largest European producer and representing 18% of European production [2].

The specialization of a small number of crops in a rotation sequence along with cultivation in greenhouses facilities and the restricted or limited use of fumigants and chemical plant protection products have increased the occurrence of plant parasitic nematodes, especially root-knot nematodes (RKN)*, Meloidogyne* spp., in vegetable production [3]. In particular, *M. arenaria*, *M. incognita*, and *M. javanica* are the most common RKN species affecting and damaging crops in the Mediterranean basin, where the climate and agronomical practices are favorable for nematode population build-up [1]. Maximum yield losses caused by RKN in greenhouse crops ranged from 37% in watermelon to 88% in cucumber [4,5]. In common beans, RKN produce nutritional deficiencies, which cause common beans to undergo a delay in growth and cause significant yield losses [6,7,8,9].

*Meloidogyne* spp. are obligate sedentary endoparasitic nematodes and the species mentioned above reproduce parthenogenetically. The life cycle of *Meloidogyne* includes six development stages: the egg stage, four juvenile stages (J1–J4), and the adult stage. The life cycle begins when the only infective and mobile stage, J2, penetrates the roots. Then, the nematode migrates through intercellular spaces to establish a permanent feeding site in the vascular cylinder, in which it will induce the differentiation of five to seven cells, named giant cells, that experience morphological, physiological, and molecular modifications caused by the J2 infection and will supply food to the nematode. The root tissue becomes distorted due to the hyperplasia around the cell, forming the root gall. Once the nematode establishes its feeding site, it will become sedentary and will grow in length and width (J3–J4), until reaching the adult stage. In favorable conditions, J2 develop into pear-shaped females. They lay eggs into a gelatinous matrix (egg mass), generally outside of the gall. A female can lay from 500 to 1500 eggs in a single egg mass. Embryogenesis will lead to a J1 developing inside the egg which will molt to J2 that will emerge and migrate into the soil. Only males appear under unfavorable conditions, and they will migrate into the soil with no evidence of feeding in plants [10].

As poikilothermic animals, the *Meloidogyne*’s length of life cycle is influenced by soil temperature [11], which is the main abiotic factor that determines the rate of development, and it will depend on an RKN–plant species interaction [3,4,5,12,13,14,15,16,17]. The range of optimal temperatures for the development of the most frequent *Meloidogyne* spp. in vegetable crops is around 28–30 °C, whereas below 10 °C or over 35 °C, little or no activity is observed [18]. In order to know the thermal requirements of *Meloidogyne* spp. in a specific plant, two constants need to be determined: the base temperature (*Tb*), defined as the minimal soil temperature from which a given biological process begins; and the thermal constant (*S*), that is, the accumulated degree days (DD) necessary to complete a given biological process. DD are the number of degrees over *Tb* accumulated in a day. The rate of nematode development is assumed directly proportional to increasing soil temperatures over *Tb* until reaching the optimal temperature (*To*). Once *To* is exceeded, the rate of nematode development decreases until reaching the maximum soil temperature (*Tm*), over which nematode development stops. Hence, nematode development occurs between *Tb* and *Tm*. Once the values of *Tb* and *S* are determined, the duration of the nematode’s life cycle along with every nematode development stage at any soil temperature can be predicted [19].

Within the framework of the sustainable use of pesticides European directive 2009/128/EC, which enforces the replacement of chemical plant protection products by environmental and human health-friendly management techniques, deep knowledge of the *Meloidogyne* life cycle is important to develop effective and durable strategies for each agricultural condition. Thus, knowledge of the thermal requirements of *Meloidogyne* on a certain crop is the basis for constructing phenology models, which can be used for advice purposes as a decision tool to maximize the efficacy of control methods such as planting date or trap crops, among others. By modifying the planting date, the plant will escape from nematode infection at early plant stage development, which will reduce the number of generations that the nematode can complete in it and also disease severity. By using trap crops, the nematode can infect plant roots but it will not reproduce by the end of the crop if it is finished before achieving *S*, and consequently, the nematode densities will be reduced for the following crop.

The creation of a phenology model begins with the estimation of *Tb* and *S* for specific nematode species–plant species interactions in a set of constant soil temperatures. Afterwards, the model has to be validated in field conditions, in which fluctuating soil temperatures occur, and where soil temperatures under *Tb* and/or over *To* can take place. Then, the calculation of DD should be adjusted to be consistent with the observed nematode development. Different methods to calculate DD have been described—the average daily temperature–*Tb,* the single or double sine, or the single or double triangle—but none of them consider the effect of soil temperatures over *To* on nematode development. The sine method simulates a sine curve with the daily minimum and maximum temperatures and then calculates DD as the area above *Tb* and below the curve. It describes the daily temperature cycle more realistically [20]. Once the area has been calculated, an adjustment of the area above *To* has to be performed because the rate of development can be negatively affected over this temperature. Then, three cutoff methods have been proposed for DD calculation adjustment—horizontal, intermediate, or vertical—depending on if the development continues at the same rate than at *To,* it is slower, or it is stopped, respectively [21]

Phenology models of *Meloidogyne* spp. on some of the most important Mediterranean vegetable crops have been developed, such as for *M. ethiopica* [15], *M. hapla* [12], *M. hispanica* [16], *M. incognita* [13], and *M. javanica* [22] on tomato; for *M. hapla* on cabbage [12]; for *M. incognita* and *M. javanica* on cucumber, zucchini squash, and watermelon [4,5,17]; and for *M. arenaria* on melon [14], but as far we know, there are not any for *Meloidogyne* spp. on common bean. Therefore, the present study aimed to determinate the thermal requirements of *M. incognita* in *P. vulgaris* to create a temperature-based phenology model and to validate it at fluctuating soil temperatures in a pot experiment in a glass greenhouse and in a plastic greenhouse at four different planting dates.

## 2. Results

### 2.1. Thermal Requirements of M. incognita on P. vulgaris.

The average soil temperatures and the range of variation in each growth chamber in the first experiment were 17.2 ± 0.7, 21.3 ± 0.9, 26 ± 1.3, 27.2 ± 0.7 °C, and 17.5 ± 2.7, 22 ± 1.1, 24.9 ± 0.8, and 27.5 ± 1 °C in the second one. The number of days needed by *M. incognita* to complete a given period of the *M. incognita* life cycle (PLC) in *P. vulgaris* at different average soil temperatures in each experiment is shown in Table 1.

**Figure 1:**
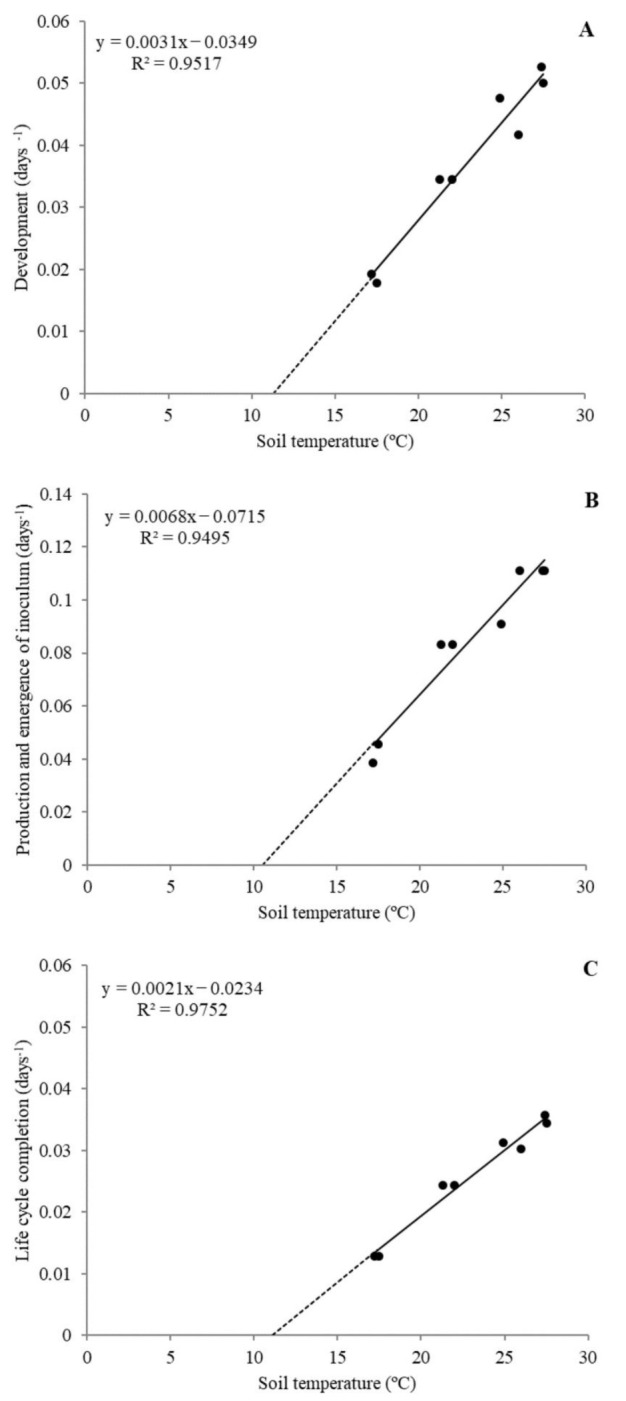
Linear regression between soil temperature (°C) and the inverse of the time (days^−1^) for: (**A**) development, (**B**) production and emergence of inoculum, and life cycle completion of *Meloidogyne incognita* in *Phaseolus vulgaris* cv. Enana Nassau.

No differences were found between experiments (*p* < 0.05), allowing the data to be pooled and a general model to be built for each of the three PLC studied (Figure 1). Thermal requirements from J2 inoculation to female starting laying eggs (development) were *Tb* =11.3 and *S* = 323 DD (Figure 1A); from development to J2 emergence (production and emergence of inoculums) were *Tb*= 10.5 °C and *S* = 147 DD (Figure 1B); and for life cycle completion *Tb* = 11.1 and *S* = 476 DD (Figure 1C). The slope of the regression lines differed between PLC (*p* < 0.05) but only the *Tb* for the production and emergence of inoculums differed from the remaining PLC (*p* < 0.05); the following validations of PLC development and life cycle completion were realized with *Tb* = 11.2 °C.

### 2.2. Validation of the Phenology Model of M. incognita on P. vulgaris at Fluctuating Soil Temperatures in Glass Greenhouse

During the pot test experiment, minimum soil temperatures lower than *Tb* for life cycle completion were registered in 4 out of 43 days, ranging from 8.8 to 10.4 °C. The maximum soil temperatures higher than *To* were registered in 27 out of 43 days, ranging from 30.1 to 35.7 °C. On April 19th, two gelatinous matrices without eggs were observed, but on the 22nd of April, from a total of 60 gelatinous matrices, 15 egg masses with an average of 11 eggs per egg mass were recorded. Then, the 20th of April was considered as the starting date of egg production for the phenology model’s validation. The life cycle was completed on the 30th April, 43 days after nematode inoculation, when 2 empty eggs were observed from more than 8000 eggs per plant in average. 

**Figure 2:**
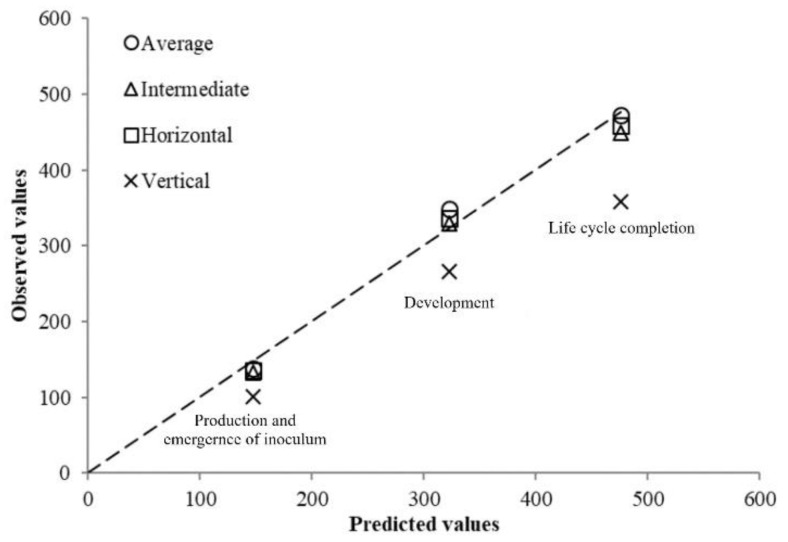
Observed versus predicted values of the accumulated degree day (DD) for development, production and emergence of inoculums, and life cycle completion of *M. incognita* on *P. vulgaris* in glass greenhouse pot test at fluctuating soil temperatures calculated with four different methods: average daily temperature-*Tb*, and single sine over *Tb* using intermediate, horizontal, and vertical cutoffs.

The relationship between the predicted and the observed results according to the different accumulated DD calculation methods is presented in Figure 2. The average daily temperature-*Tb,* along with the single sine method with horizontal or intermediate cutoff were the most accurate methods of DD calculation (93–106% of the predicted value) whilst the single sine method with vertical cutoff underestimate the DD calculation (75–82% of the predicted value).

### 2.3. Validation of the Thermal Requirements of M. incognita on P. vulgaris at Fluctuating Soil Temperatures in Plastic Greenhouse.

The soil temperatures during the common bean crops’ transplantation on four different dates ranged between 14.6 and 30.7 °C. The maximum soil temperature over 30 °C was recorded on only one day during the fourth common bean crop, and then, soil temperatures were considered to be in the range between *Tb* and *To*. Consequently, the DD calculation was carried out only by the average daily temperature–*Tb* method. Life cycle completion was observed between 453 and 476 accumulated DD with accuracy between 0.95 and 0.99 (Table 2). The nematode’s life cycle was completed 47, 46, 42, and 40 days after transplanting each crop, when a number of empty eggs ranging from 1 to 28 were observed among 7000 eggs.

## 3. Discussion

The present study provides novel information concerning the thermal requirements of *M. incognita* on *P. vulgaris* to construct a temperature-based phenology model for predicting the rate of progression of the disease cycle considering three relevant periods: from transplanting to female starting to lay eggs, egg hatching, and life cycle completion. Moreover, the accuracy of different degree day calculation methods has also been assessed in order to be used for disease management purposes. 

**Figure 3:**
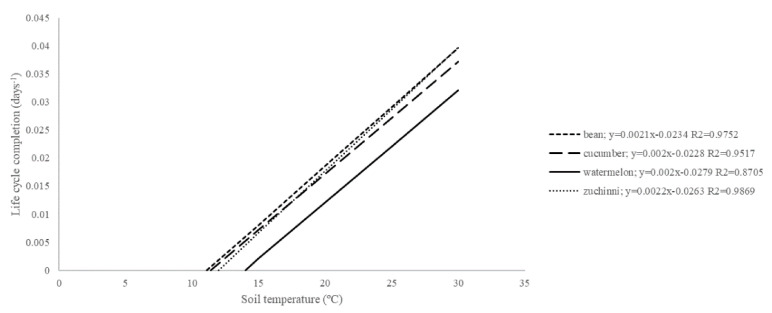
Relationship between soil temperature and the life cycle development rate of the isolate Agròpolis of *Meloidogyne incognita* on cucumber [4], watermelon [5], zucchini squash [17], and on common bean (current study).

The thermal requirements of each PLC of *Meloidogyne* depend on the plant host and the nematode species. In fact, the thermal requirements of the *M. incognita* isolate used in the current study to complete its life cycle on *P. vulgaris* (*Tb* = 11.1 °C; *S* = 476 DD) were similar to those obtained for the same nematode species in zucchini squash (*Tb* = 12 °C; *S* = 455 DD) [17] and cucumber (*Tb* = 11.4 °C; *S* = 500 DD) [4] but differed from the one on watermelon (*Tb* = 14 °C; *S* = 500 DD) [5] (Figure 3). It is accepted that watermelon is a poor host of *Meloidogyne* [5,23]. That is, this plant species is less suitable for increasing nematode densities than others for a given period of time, as the phenology model shows. The *M. incognita* isolate Agròpolis needs higher *Tb* and needs to accumulate more DD for its life cycle completion on watermelon than on *P. vulgaris* and other cucurbit crops. Additionally, the thermal requirements can vary between *Meloidogyne* species for a given plant species, as has been reported by different authors on tomato (Table 3). On potato, Charchar and Santo [24] reported similar *S* for the races 1 and 3 of *M. chitwoodi* to complete a life cycle at almost all temperatures tested, but different from the ones of *M. hapla*. Similarly, different *S* and *Tb* of *M. incognita* and *M. javanica* were reported for all PLC on watermelon [5], or from J2 to egg-laying females of *M. arenaria*, *M. incognita*, and *M. javanica* on okra [25]. Nonetheless, in most of these studies, the obtained regressions were not statistically compared in order to construct a general model to facilitate its use for advisement purposes. When regression comparisons were performed, no differences were found in the thermal requirements for *M. incognita* and *M. javanica* development, egg hatching, and life cycle completion on cucumber or zucchini squash [4,5], allowing the constructing of general phenology models. Information on the thermal requirements of *Meloidogyne* spp. on *P. vulgaris* is scarce. Strajnar et al. [15] studied the *M. luci* (previously reported as *M. ethiopica*) thermal requirements for nematode development (inoculation to female starting to lay eggs) on different crops, including common bean at fluctuating soil temperatures. These authors observed nematode eggs 51 and 24 days after nematode inoculation of common beans at average daily temperatures of 18.3 °C (15–21 °C) and 22.7 °C (20–25 °C), respectively. Unfortunately, these authors only obtained results from two out of the three temperatures assessed because at the higher temperatures assessed (26.3 °C; 22–30 °C), plants did not develop properly. According to our phenology model for this PLC, conducted at constant temperatures, egg production should be started at 56 and 28 days after nematode inoculation. Therefore, the accuracy of our phenology model was 91 and 86% of the predicted data, respectively, insufficient to be used for advisory purposes.

During the validation of the phenology model in the pot experiment in the glass greenhouse, soil temperatures suffered large fluctuations, being lower than the *Tb* determined in the growth chamber and higher than the *To* for each PLC. The accumulated degree day calculation for each observed event was conducted using four different methods: the average daily temperatures method–*Tb* (ADTb) and the single sine method over *Tb* (SSTb) with three different cutoffs (horizontal, intermediate, and vertical) and their accuracy estimated with regard to the thermal requirements was obtained from the growth chamber experiments. The ADTb and the SSTb with horizontal or intermediate cutoff calculation methods were the most accurate ones for nematode development, egg hatching, and life cycle completion, whereas the SSTb with vertical cutoff underestimated the accumulated DD because when the temperature was above *To*, the latest method ruled it out. The validation of the thermal requirements in the plastic greenhouse confirmed the ones generated in the growth chamber, with small differences using the ADTb. This system was used because the soil temperatures recorded during the four planting dates were no lower than the calculated *Tb* in the growth chamber experiments and no higher than *To.* Therefore, in our agronomic conditions, where soil temperatures at 15 cm depth in the greenhouse are only above *To* on very few days at the end of horticultural cultivated in winter-summer or spring-summer seasons [26,27], and the range of temperatures of common beans are between 10 and 30 °C [28,29], the ADTb is the most appropriate because it is the easiest calculation method. However, the most suitable accumulated DD calculation method should be determined for any cropping area for improving the accuracy of the phenology model.

Knowledge of the thermal requirements of *Meloidogyne* species on specific plant species, and the validation of the constructed phenology models in specific growing areas, allows prediction of the time to reach the most vulnerable PLC and to design nematode management strategies for improving its efficacy: for example, to decide the plant material according to the length of its cycle, the sowing or transplanting date, the use of trap crops, or nematicide application. The common cv. Enana Nassau has a short cycle compared to other common beans, producing after 50 days of sowing when cultivated between February and November according to the seed company. However, in the agricultural conditions of our experiments, the crop was cultivated in spring at soil temperatures between 14.6 and 30.7 °C, being within the range of common bean development temperatures [28,29], and the nematode completed its life cycle before crop yield began (47, 46, 42, and 40 days after transplantation). Consequently, nematode densities at the end of the crop will increase, compromising the performance of the following crop in the rotation sequence. The selection of shorter cycle common bean cultivars could allow completing of its cycle before nematode reproduction, acting as a trap crop. Another possibility could be cropping the common bean cv. Enana Nassau in autumn, when soil temperatures become colder, allowing nematode infection (soil temperatures over *Tb*) but not reproduction (accumulated DD lower than *S* for female starting-laying eggs) because of a reduction in the rate of nematode development, being a trap crop, as was observed with lettuce transplanted in October or November, but not in September, in a plastic greenhouse in northeastern Spain [1]. The modification of the planting date can also have an effect on crop yield losses. For instance, by growing common bean from September to December in Egypt, *M. arenaria* could cause yield losses but not when it was cultivated from February to May, when the soil temperatures were lower [8]. At the same time, the early nematode infection would be skipped, and the number of nematode generations would be reduced along with disease severity and crop yield losses. Regarding nematicide application, it is necessary to know what nematode development stages are affected, and how long it is active to determine when it should be applied. If biological-based nematicides are used, the thermal requirements of the biological control agents must also be known to determine when they should be applied in order to allow enough growth to maximize their effects when the most vulnerable nematode stage appears. Thus, phenology models can be used as a tool to make decisions for maximizing the efficacy of several control methods used in integrated nematode strategies fitting in the European Directive 2009/128/EC, willing to preserve and guarantee a positive contribution to the agricultural sector, environment, and human health.

## 4. Materials and Methods 

### 4.1. Thermal Requirements of M. incognita on P. vulgaris

Seeds of common bean cv. Enana Nassau (Semillas Batlle, Molins de Rei, Spain) were sown in 200 cm^3^ pots with sterilized river sand (1 h at 121 °C, procedure repeated after 24 h). Then, pots were placed in a growth chamber at 25 ± 3 °C with a 16:8 h light:dark photoperiod. After 10 days, 40 plants per temperature were placed in growth chambers at four different constant soil temperatures and at a 16:8 h light:dark photoperiod. Plants were acclimatized in each growth chamber for two days before nematode inoculation. At sowing, slow-release fertilizer was applied on the soil surface (Osmocote plus ©, Scotts Company LLC, Marysville, OH, USA: NPK 15-9-12 + 2% MgO + microelements). Irrigation was performed manually with water maintained in each chamber to avoid temperature fluctuations. The frequency of irrigation varied according to the water needs of each plant conditioned by the growth chamber temperature. Soil temperatures were recorded in each growth chamber at 30 min interval with a probe (PT100, Campbell Scientific Ltd. ©, Loughborough United Kingdom) at 4 cm depth in the pots. 

The *M. incognita* isolate Agròpolis maintained in susceptible tomato (*Solanum lycopersicum*) cv. Durinta (Seminis Seeds, St. Louis, Missouri USA) was used in this study. The nematode species was identified by perineal patterns and molecular SCAR–PCR markers [30]. The nematode inoculum used consisted of second-stage juveniles (J2) that emerged from eggs extracted from tomato roots through Hussey and Barker’s method [31] by blender maceration in a 5% commercial bleach (40 g L^−1^ NaOCl) for 10 min. The egg suspension was passed through 75 µm-pore sieve to remove organic material and then passed through a 25 µm pore sieve to retain the eggs, which were placed in a Baermann tray [32] for two weeks at room temperature. J2 from the first 24 h were discarded. Afterwards, J2 were collected every 48 h for 10 days, counted, and maintained at 9 °C until inoculation.

Three weeks after sowing, plants were inoculated with the *M. incognita* population Agròpolis at a rate of 1 J2 per cm^3^ of soil. The nematode inoculum was added into two holes in the soil at 1 cm from the stem and at 3 cm of depth. Periodically, nematode development was assessed in three plants grown at each temperature. Nematodes were stained inside the roots with acid fuchsine (Panreac Quimica SA Castellar del Vallès Spain) [33]. When females were observed inside the roots, egg production and egg hatching were evaluated by washing the roots with tap water and then submerging them into a 0.1 g L^−1^ erioglaucine solution (Acros Organic, Geel, Belgium) to stain the egg mass blue [34]. Afterwards, roots were washed with tap water and single egg masses were handpicked and deposited in entomological vials containing a 10% bleach solution (40 g L^−1^ NaOCl) for egg disaggregation from the egg mass to observe egg development stages and the presence of J2 and/or the first empty egg. The experiment was repeated once.

The average soil temperature in each growth chamber was obtained for *Tb* and *S* estimation for each of the three periods of the *M. incognita* life cycle (PLC): from nematode inoculation to females starting to lay eggs (Development), from egg production to the emergence of J2 (Production and emergence of inoculum), and from inoculation until the emergence of J2 (Life cycle completion). The days elapsed in each period were expressed as the reciprocal of time to construct a lineal regression with the temperature of each growth chamber. Regression was analyzed to estimate *Tb* as the soil temperature at which the reciprocal of time = 0 (reciprocal of time = *aT−b; Tb = b/a*; intercept), and *S* (inverse of the slope) [35]. The regressions of each period were contrasted between experiments by the proc glm procedure using SAS v. 9 (SAS Institute Inc., Cary, NC, USA) and if no significant differences were found (*p* < 0.05), the data were analyzed together to create a general model. In addition, the regression lines of each PLC were contrasted between them to determine differences in *Tb* and between *S.*

### 4.2. Validation of the Phenology Model of M. incognita on P. vulgaris at Fluctuating Soil Temperatures in Glass Greenhouse

The validation of the phenology model at fluctuating soil temperatures was carried out from 12th March to 30th April in a pot test in a glass greenhouse located in Viladecans (Barcelona, Spain) of the UPC. 

Forty-one-month-old plants of the common bean cv. Enana Nassau were transplanted in 1 L pots with sterile river sand and were placed on glass greenhouse benches. Plants were fertilized with slow release fertilizer (Osmocote plus ©, Scotts Company LLC, Marysville, OH, USA) and irrigation dose and frequency were adjusted to crop needs. Soil temperatures were recorded at 8 cm depth at 1 h intervals with 5 TM probes (Decagon Devices Inc. ©, Pullman, WA, USA) located in four different pots. After one week (19 March), the soil was infested with the *M. incognita* isolate Agròpolis at a rate of 1 J2 cm^−3^ of soil.

The evaluation frequency of nematode development was conditioned by the accumulated soil temperatures related to the thermal requirements obtained at constant soil temperatures in growth chambers. When a given PLC was expected to occur, two plants were removed and processed by staining the nematodes inside the roots with acid fuchsine [33] or the egg masses in an erioglaucine solution [34] as previously stated.

The accumulated DD were calculated by the average of daily soil temperatures–*Tb* (ADTb) as well as using the Degree Day calculator from the University of California [36] with the single sine method over *Tb* (SSTb) model with each of the three cutoff methods: horizontal, intermediate, and vertical [21]. The *To* was considered 30 °C and the lower threshold was the *Tb* for each PLC obtained from growth chamber experiments. The accuracy of each DD calculation method was estimated by the relationship between the observed and the predicted data for each PLC.

### 4.3. Validation of the Thermal Requirements of M. incognita on P. vulgaris at Fluctuating Soil Temperatures in Plastic Greenhouse.

The experiment was carried out in the plastic greenhouse of the UPC located in Viladecans (Barcelona, Spain), from 3 March to 5 May. The soil texture was sandy loam, with 83.8% sand, 6.7% loam, and 9.5% clay; pH 8.7; 1.8% of organic matter (*w/w*) and 0.5 dS m^−1^ electrical conductivity. The soil had been artificially infested with the *M. incognita* isolate Agròpolis in 2007. Four plots of 9.6 m^2^ each were used in the experiment. The initial nematode density (*Pi*) in each plot was determined by taking 8 soil cores collected with an auger (30 cm high and 2.5 cm diameter). The soil was mixed, passed through a 4-mm sieve to remove stones, and nematodes were extracted from a subsample of 500 cm^3^ of soil by Baermann trays [30] incubated at room temperature for a week. Afterwards, J2 were collected in a 25 µm-pore sieve and then counted.

Lots of 50 seeds were sown each week for four consecutive weeks in a mixture of peat and vermiculite substrate one month before being transplanted. Plantlets were transplanted the 3rd, 10th, 18th, and 24th of March, in order to achieve differences in soil temperatures. In each plot, plants were spaced 50 cm between rows and 55 cm within rows. Plants were fertilized weekly with NPK solution (15-5-30) at 31 kg ha^−1^ and micronutrients at 0.9 kg ha^−1^, supplied through a drip irrigation system. Soil temperatures were recorded at 15 cm depth at one-hour intervals with three 5TM probes (Decagon Devices Inc. ©, Pullman, WA, USA). Plants were uprooted periodically for assessing nematode development into the roots according to the procedure described previously. The assessment frequency was increased when the life cycle was near completion. The calculation of the accumulated DD and the relationship between the observed and the predicted data was carried out as previously stated.

## Figures and Tables

**Table 1:** Number of days elapsed from *Phaseolus vulgaris* cv. Enana Nassau inoculation with 1 J2 cm^−3^ of soil of *Meloidogyne incognita* to female starting laying eggs (Development), from egg production to the emergence of J2 (Production and emergence of inoculum), and from inoculation to the emergence of J2 (life cycle completion) in pots at constant soil temperatures.

Repetition	Soil Temperature (°C)	Period of the *M. incognita* Life Cycle (PLC)		
		Development (days)	Production and Emergence of Inoculum (days)	Life Cycle Completion (days)
1	27.4	19	9	28
	26.0	24	9	33
	21.3	29	12	41
	17.2	52	26	78
2	27.5	20	9	29
	24.9	21	11	32
	22.0	29	12	41
	17.5	56	22	78

**Table 2:** Accumulated degree days (DD) for life cycle completion of *Meloidogyne incognita* on *Phaseolus vulgaris* cv. Enana Nassau observed at four planting dates in a plastic greenhouse (Viladecans, Spain) at alternating soil temperatures between *Tb* and *To* calculated with the average daily temperature–*Tb* method and that obtained by linear regression at constant soil temperatures in growth chambers (predicted) and the estimated in laboratory and the observed versus estimated values comparison.

Planting Date	Accumulated DD		Observed/Predicted
	Observed	Predicted	
3 March	453	476	0.95
10 March	472		0.99
18 March	456		0.96
24 March	463		0.97

**Table 3:** Base temperature (*Tb,* °C) and accumulated degree days over *Tb* (*S*, °C) (*S*) for life cycle completion of different *Meloidogyne* species on tomato.

*Meloidogyne spp.*	*Tb*	*S*	References
*M. arenaria*	10.3	313	[16]
*M. hapla*	8.3	553	[12]
*M. hispanica*	10.4	526	[16]
*M. incognita*	10.1	400	[13]
*M. javanica*	12.9	350	[22]

*Tb* is the base temperature defined as the minimal soil temperature from which a given biological process begins and *S* is the thermal constant defined as the accumulated degree days over *Tb* necessary to complete a given biological process.

## Data Availability

Data sharing not applicable.
